# A design-integrated framework for neuroarchitectural research

**DOI:** 10.3389/fpsyg.2026.1666480

**Published:** 2026-02-13

**Authors:** Na Wei, Erick Gustavo Chuquichambi, Claudia Damiano, Keaton Bruce, Dirk B. Walther

**Affiliations:** 1 Tyler School of Art and Architecture, Temple University, Philadelphia, PA, United States; 2 Department of Psychology, University of the Balearic Islands, Palma de Mallorca, Spain; 3 Department of Psychology, University of Toronto, Toronto, ON, Canada

**Keywords:** architectural perception, curvature, embodied spatial experience, experimental framework, immersive environment, neuroaesthetics, neuroarchitecture, stimulus design

## Abstract

This paper presents a multi-stage framework that integrates architectural design logic with empirical testing across representational scales. This exploratory, practice-driven research paradigm seeks to bridge disciplinary divides between (1) empirical rigor and situated experience, (2) visual abstraction and embodied spatial engagement, and (3) neuroscientific measurement and architectural meaning. Through a systematic reassessment of dominant methodological assumptions in current neuroaesthetics and neuroarchitectural studies, with curvature serving as a test case, the paper develops a three-phase experimental approach comprising image-based evaluation (Phase 1), object-based interaction (Phase 2), and full-scale immersive experience (Phase 3), supported by a stimulus-generation pipeline rooted in spatial logic and architectural composition. The paper details the design rationale and implementation of stimuli across all phases. Findings from Phase 1 are briefly presented to (i) illustrate the framework's capacity to generate perceptually distinct stimuli and reveal recurring evaluative patterns, including foreground-sensitive preferences for curvature under controlled figure–ground manipulations; and (ii) inform the methodological calibration and design logic of subsequent Phase 2 and Phase 3 investigations, which are currently ongoing and are presented in terms of methodological structure and exploratory potential. Rather than treating the architectural environment as a passive object of evaluation, the framework positions it as a generator of perceptual, affective, and behavioral data within an iterative research process. Scalable and adaptable, this approach supports collaborative investigations across multiple contexts and offers a pathway for advancing neuroarchitectural research and progressively increasing ecological validity. Several key terms are employed in their architectural sense to support cross-disciplinary dialogue.

## 1 Introduction

Advances in scientific technology have led to the development of tools that enable empirical approaches in neuroscience and cognitive science (Zeki, 1998). These methods have produced a growing body of research on perception, emotion, and cognition, and have more recently been extended to the study of the built environment, particularly with respect to the neural and affective responses to architectural form (Edelstein, 2015; Coburn et al., 2017; Chatterjee et al., 2021). This trajectory has contributed to the emergence of the interdisciplinary field of neuroarchitecture, which applies empirical methods to examine how spatial design influences emotional and behavioral outcomes (Ekman and Davidson, 1994; Davidson and Milligan, 2004; Micieli-Voutsinas and Person, 2020; Farahi, 2020). Driven primarily by scientific methodologies, much of this work has been led by scholars in neuroscience and psychology and has therefore developed largely outside the disciplinary logics of architectural theory and practice.

Despite growing interest among architectural researchers, the discipline has remained cautious about engaging with neuroscientific findings. This hesitation reflects deeper concerns regarding methodological incompatibilities between neuroscientific experiments and the epistemological foundations of architectural knowledge (Pérez-Gómez, 1983; Gepshtein and Snider, 2019; Ritchie, 2020; Zisch, 2020; Lee et al., 2022; Wang et al., 2022; Magsamen et al., 2023). At the crossroads of art, science, and philosophy, architecture is grounded in a disciplinary tradition organized around three core values: durability or buildability, utility or functionality, and beauty or delight (Vitruvius, 1999). These values are apprehended through embodied, multisensory, and temporally extended experience rather than isolated perceptual moments (Giedion, 1982; Norberg-Schulz, 1980; Mallgrave, 2015).

From an interdisciplinary perspective, this paper argues that many current methods in neuroarchitecture encounter recurring and interrelated limitations. Acknowledging that architectural knowledge is accumulated through design-experience iteration, this paper proposes a design-integrated experimental framework with a multistage methodological structure that aligns the compositional workflows of architectural practice with neuroscientific accounts of how environments are processed across representational and immersive modalities (Landau and Jackendoff, 1993; Kozhevnikov and Dhond, 2012; Djebbara and Kalantari, 2023).

This orientation positions the study not as a test of one-to-one correlations, but as a methodological inquiry within an exploratory paradigm, aimed at bridging three key disciplinary divides: (a) empirical rigor and situated experience, (b) visual abstraction and spatial engagement, and (c) neuroscientific measurement and architectural meaning.

## 2 Critique: a case study of curvature-based neuroarchitectural research

To enable experimental control, many empirical studies in neuroaesthetics rely on simplified, decontextualized stimuli and narrowly defined behavioral tasks. While such approaches yield provocative findings, they often reduce architectural experience to isolated attributes, offering only a partial account of how architecture is perceived, inhabited, and evaluated in practice (Saidi, 2019; Spence, 2020).

Moreover, much of this literature focuses on responses to completed forms or their representations (e.g., photographs), with limited attention to the design process through which spatial, material, and organizational attributes are shaped. This omission of design reasoning contributes to a widening gap between experimental control and architectural relevance, increasing the risk of reductionism.

To examine these tensions, this paper revisits methodological practices common in neuroaesthetics—and more specifically in neuroarchitecture—through a case study of the highly cited work by Vartanian et al. (2013). This study reported enhanced aesthetic preference and positive emotional responses to curvilinear architectural interiors and has been widely cited and extended (Gómez-Puerto et al., 2016; Banaei et al., 2017; Shemesh et al., 2022; Tawil et al., 2024), amplifying its influence across disciplines. Although some of its limitations have been acknowledged within the neuroarchitecture literature, existing critiques remain partial and unsystematic and have not prompted *a substantive methodological reorientation*. A more comprehensive reassessment is therefore needed—not to diminish its contribution, but to clarify its scope and prevent its findings from becoming uncritically generalized.

More broadly, the case of curvature research reveals a structural tension between neuroscientific experimental paradigms and architectural phenomenology. This tension arises when experimental designs insufficiently engage architectural knowledge, spatial reasoning, and design logic from the outset. The foundational studies, such as Vartanian et al. (2013), provide valuable insights into inherent challenges that the emerging field of neuroarchitecture faces, and serve as a point of departure for developing empirical methodologies that are more closely aligned with architectural practice and theory.

The following analysis is organized around four methodological limitations identified in current neuroaesthetic and neuroarchitectural research.

### 2.1 Stimuli reductionism: conceptual and temporal flattening

A primary limitation of early empirical research on the perception of architecture lies in its treatment of static decontextualized images as adequate proxies for architectural space. This methodological reduction flattens architectural experience both representationally and conceptually, overlooking spatial volume, material presence, multidirectional enclosure, and embodied affordances-qualities fundamental to how architectural environments are perceived and inhabited. When isolated visual features are treated as sufficient stimuli, architectural form is effectively abstracted from its spatial, sensory, and functional contexts. Consequently, the reported preference for curvilinear forms reflects judgments about pictorial representations rather than inhabitable environments, resulting in an unsubstantiated shift in the object of judgment.

Temporal flattening further compounds this limitation. Practical constraints common in fMRI protocols, such as brief stimulus presentations (e.g., approximately 3 s), prioritize momentary neural responses over the extended temporal unfolding characteristic of real-world spatial encounters. Such compression constraints exclude dwelling, memory accumulation, attentional shifts, and emotional resonance, which are central to architectural experience, long emphasized in architectural theory (Bachelard, 1994; Heidegger, 1996; Pallasmaa, 2012). These considerations highlight the need for experimental approaches that better accommodate the *durational* and *reflective* dimensions of inhabiting space.

### 2.2 Semantic inconsistency: uncontrolled variability and typological bias

The second critique centers on the semantic inconsistency of the stimuli. For example, Vartanian et al. (2013) study used (a) *found images* from architectural databases, (b) two architects as the raters, and (c) classified these images into “curvilinear” and “rectilinear” groups. However, the selected interiors vary substantially in design intent, spatial program, material articulation, and construction resolution. Many curvilinear examples depict expressive, high-design interiors, whereas rectilinear examples depict more utilitarian or conventional settings (Vartanian et al., 2013). This typological imbalance risks conflating contour with broader cues of design quality and aesthetic capital (Vitruvius, 1999; Rowe and Slutzky, 1963).

The instability of this curvilinear–rectilinear classification further compounds this issue. The two categories are derived from expert judgments of perceived contours in photographs, rather than from measurable properties of the underlying spatial geometry (Chuquichambi et al., 2022). Participants, by contrast, evaluate the same images by imaginatively reconstructing space through everyday experience, memory, and cultural association. Prior work also shows that experts and non-experts can diverge in aesthetic judgments (Cotter et al., 2017; Vartanian et al., 2019; Corradi et al., 2020; Palumbo et al., 2022). Consequently, the relationship among designed geometry, photographed representation, and lay reconstruction remains indeterminate, limiting the interpretability and reproducibility of curvature effects.

This semantic ambiguity also reflects a broader methodological lineage. Bar and Neta (2006) contrasted curvilinear and angular objects in 2D images and reported a general preference for curvature. Vartanian et al. (2013) extended this image-based approach to photographs of architectural interiors, substituting *rectilinearity* for *angularity*—a shift that changes the comparison from perceptual extremes to a contrast between conventional and non-conventional design typologies, thereby embedding semantic and aesthetic biases into the experimental logic itself.

A follow-up study by Vartanian et al. (2024) offers a more cautious framing, emphasizing that curvature perception depends on higher-order, context-sensitive mechanisms and underscoring that conceptual and operational concerns remain about the clarity and stability of the “curvilinear” versus “rectilinear” distinction within this experimental context.

### 2.3 Fragmented cognition: decoupling experience and response

In many neuroaesthetics studies, including Vartanian et al. (2013), behavioral responses, such as approach/avoid judgments, are collected through discrete, survey-based, forced-choice tasks rather than through action-oriented engagement. When static, decontextualized images are paired with abstract response formats, perceptual evaluation is effectively decoupled from embodied spatial experience. Participants' “enter” or “exit” decisions are therefore likely speculative and symbolic, detached from genuine spatial inclination.

In real-world architecture, perception and action are tightly intertwined; spatial preference emerges through embodied engagement, affective orientation, and perceived affordances rather than isolated visual appraisal (Spence, 2020; Djebbara and Kalantari, 2023). By constraining response to disembodied judgments, such experimental designs fragment cognition, separating what is seen from how space invites action. This limitation is underscored by a recent follow-up study, which reports a neural dissociation between predicted beauty ratings and approach–avoidance behavior, indicating that visual preference derived from static images may not reliably predict spatial desirability (Vartanian et al., 2024).

### 2.4 Ecological validity gap: generalizing experimental findings into design prescription

Beyond the structure of experimental inputs, the translation of empirical findings into architectural design presents a further methodological challenge. Scientific results are often interpreted as objective and generalizable truths; within neuroaesthetics, reported preferences—such as those favoring curvilinear forms—can therefore be overextended and implicitly elevated to design prescriptions. This raises a critical concern regarding the constraint inherent to experimental reach.

Empirical rigor denotes a specific methodological orientation characterized by variable control, reproducibility, conditional isolation, and reduction as method. It does not claim to provide a fully representative account of situated experience, which is inherently complex, contextual, and irreducible (Deleuze and Guattari, 1987). Empirical rigor and situated experience, therefore, are non-isomorphic and operate at different methodological resolutions that are not yet systematically connected.

Consequently, the pursuit of a direct equation between a single architectural attribute and observers' perceptual responses may be less insightful than the identification of recurring perceptual-experiential patterns that emerge across varied situations. The notion of *pattern*, as opposed to isolated stimulus–response regularities, refers to relational configurations between spatial form, perception, and use, most prominently articulated in Alexander et al. (1977) *A Pattern Language*. Generating such patterns requires the accumulation of situated evidence over time, as well as methodological pathways capable of scaling inquiry beyond isolated laboratory conditions. Without mechanisms that reconnect empirical findings to architectural design processes, experimental approaches risk remaining analytically precise yet practically remote. Addressing ecological validity requires not stronger causal claims, but differently structured methodological frameworks—ones that remain open to complexity, context, and iterative interpretation.

## 3 From critique to method

Building on the methodological limitations identified above, this study advances an exploratory, design-integrated experimental framework for investigating architectural curvature across progressively richer representational contexts: moving from image-based evaluation (Phase 1), to object-based interaction (Phase 2), and full-scale and immersive environments (Phase 3).

Rather than adopting a confirmatory hypothesis-testing pipeline to establish one-to-one causal relationship, the framework is designed to identify cross-scale patterns and alignments between perceptual evaluation, spatial affordances, and experiential engagement. Each phase expands the breadth of measurable experience by enabling additional modes of engagement and data collection, under increasingly ecologically valid conditions. Phase 1 establishes a controlled visual reference for perceptual calibration, and image-based survey measures are retained across phases to support comparability.

**Figure 1:**
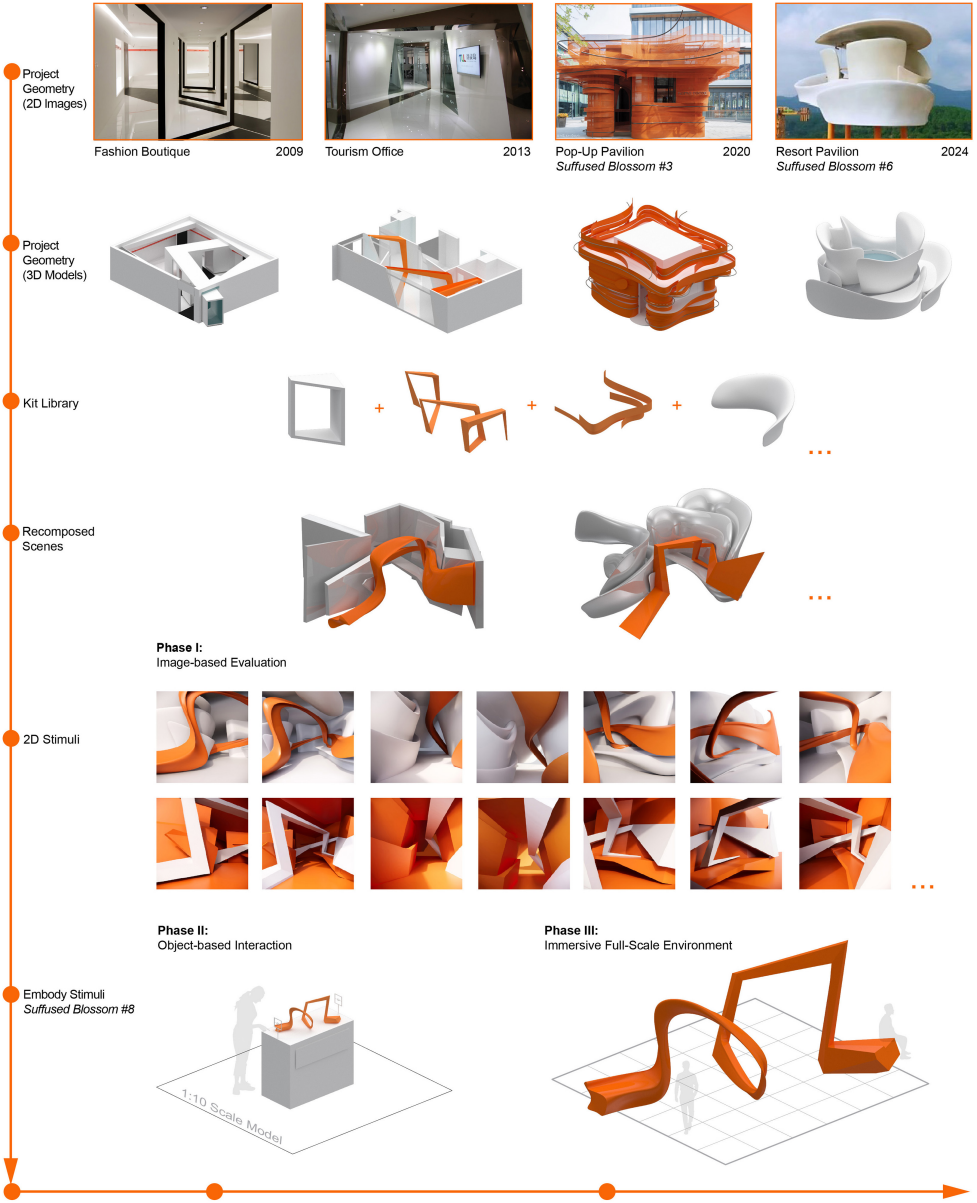
Diagram of the multi-stage, design-integrated experimental process. Architectural components digitally modeled from built works were abstracted and decomposed into points, lines, and surfaces, which are subsequently recomposed into multiple spatial scenes. The process unfolds across three representational scales: 2D rendered images used for image-based evaluation in Phase 1; 3D physical prototypes translating selected spatial configurations in Phase 2; and a full-scale immersive installation in Phase 3. The diagram presents examples of outcomes at each stage and includes human figures to indicate relative scale and to situate each representation within a consistent spatial reference.

The methodological components below are organized by operational design logic, including stimulus selection, geometric decomposition, and translation across representational media, reflecting the iterative and non-linear nature of design-led architectural research (Figure 1).

### 3.1 Project selection and geometric decomposition

To generate controlled visual stimuli while limiting stable and often implicit variations introduced by architectural design style, the study constructs a prototype corpus from approximately 150 architectural projects designed by the first author.

The selection proceeded through a two-stage filtering process. An initial screening identified projects that shared three characteristics: (a) emotional-intent-guided design approach, as defined by the emotive design methodology, which emphasizes intuition-driven formal exploration to elicit affective and experiential qualities of space (Wei, 2024; see also Wei, 2019) to acknowledge the earlier foundational work. (b) dynamic composition characterized by geometric articulation; and (c) a fully digital modeling workflow in Rhinoceros 3D, enabling systematic decomposition and recomposition.

From this subset, four projects were selected based on comparability and formal clarity, including: (1) a compact spatial scale (under 100 m^2^) to minimize variation due to size; (2) a shared cultural and programmatic context; (3) spatial function including both transitional and resting areas; and (4) a clear emphasis on either curvilinear or angular spatial form. Completed in 2009, 2013, 2020, and 2024, respectively, the selected projects exemplify four distinct geometric typologies: planar angularity (2009), volumetric angularity (2013), single-surface curvature (2020), and compound curvature (2024). Each project displays a clear figure–ground relationship, further accentuated by a high-contrast color scheme.

From these built projects, architectural elements were systematically extracted in Rhinoceros 3D through a process of geometric decomposition, focused on isolating spatial components central to architectural experience. Elements were then categorized along multiple dimensions beyond geometry: (1) *figure–ground relationships*, distinguishing foreground elements—referring to spatial components that directly engage bodily proximity and action (e.g., seating, thresholds, frames)—from background elements that establish spatial context and orientation; and (2) *functional–spatial configuration*, classifying elements as point, line, plane, or volume, understood not merely as geometric primitives but as perceptual agents that guide navigation and establish visual hierarchy, consistent with foundational architectural design methodologies (Sherwood, 1981; Ching, 2014; Hedges, 2017). For example, elongated linear volumes (lines) define spatial axes and thresholds, whereas surface-bound enclosures (planes) demarcate zones of orientation. Through this decomposition, a modular library of spatial components was established.

While component extraction followed the logic of formal clarity and spatial hierarchy, recomposition was guided by behavioral affordances and spatial use patterns through an *assemblage-based strategy*: parts are irreducibly merged into a whole (Deleuze and Guattari, 1987; Elderfield, 1992; DeLanda, 2016). These assemblies were structured around functional cues, including thresholds, bounding walls, seating surfaces, and niches.

That is, component identification was *form-driven*, whereas spatial arrangement was *use-driven*, allowing behavioral affordances to emerge from relational composition rather than isolated formal features.

As a result, seven spatial scenarios were created as 3D models, each combining compositional clarity with behavioral flow and representing spatial modes including passage, pause, or rest. Final models were hybridized, trimmed, and Boolean-simplified to ensure coherence and perceptual legibility.

### 3.2 Image-based stimuli and survey protocol

**Figure 2:**
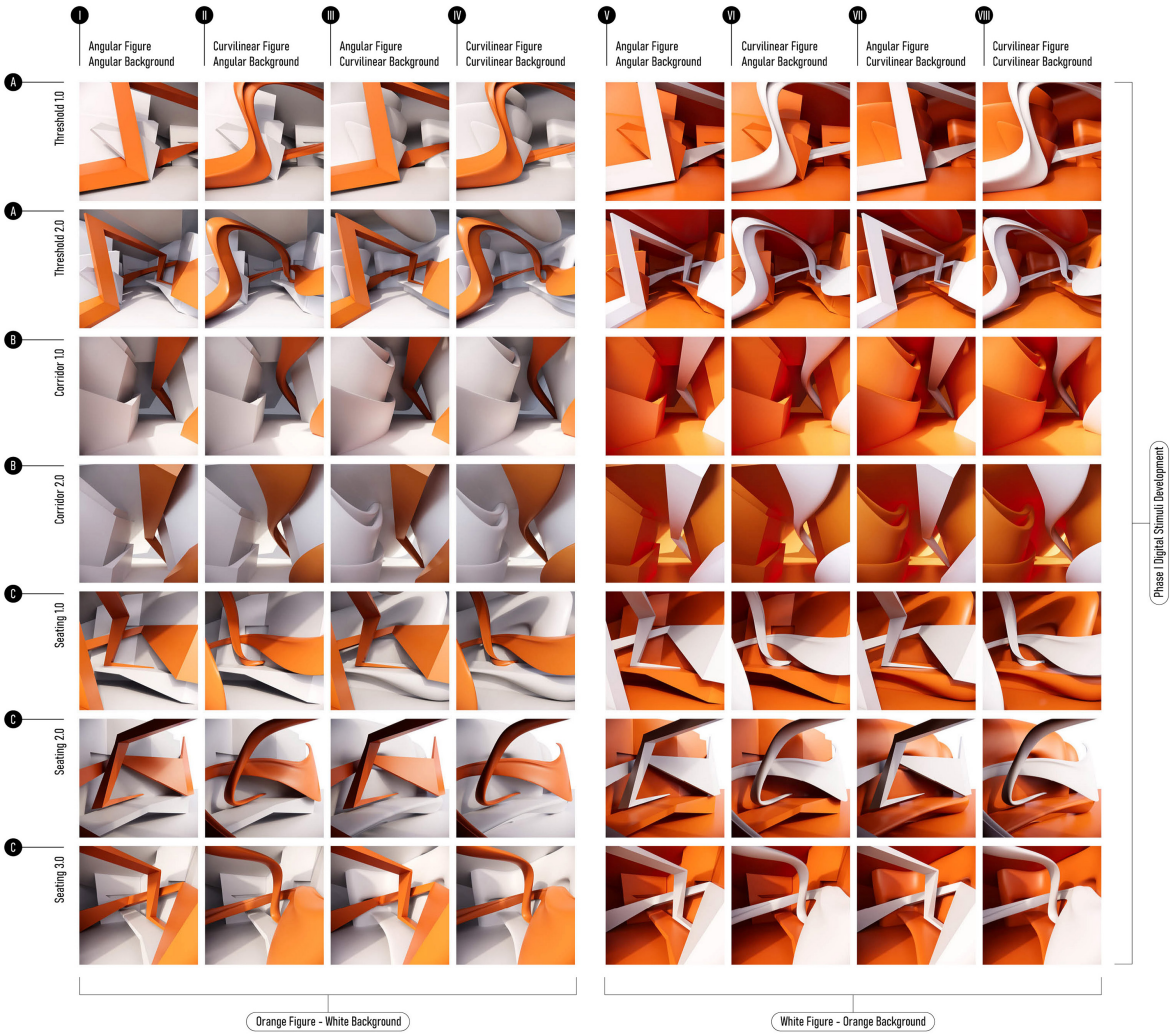
Matrix of the image-based stimulus set used in Phase 1. Seven digitally modeled spatial compositions were rendered into 56 two-dimensional images. These compositions were generated through kit-based recomposition and organized in the matrix by spatial typology and variation. Rows represent three primary spatial typologies—A: Threshold, B: Corridor, and C: Seating—with multiple versions within each typology (e.g., 1.0, 2.0, 3.0) indicating variations in spatial configuration. For each row, images are arranged according to a factorial composition varying background geometry (curved vs. angular), foreground geometry (curved vs. angular), and color (white vs. orange). Columns I–IV depict the white background condition, and columns V–VIII depict the orange background condition. Within each color group, columns are ordered by figure–background configuration: angular figure on angular background, curvilinear figure on angular background, angular figure on curvilinear background, and curvilinear figure on curvilinear background. All stimuli were rendered using standardized lighting, material, and camera parameters.

The seven spatial scenarios were then rendered as a series of controlled images that served as the Phase 1 stimuli. Each scenario was presented in eight variations −2 (Background: curved vs. angular) × 2 (Foreground: curved vs. angular) × 2 (Color: white vs. orange)—yielding 56 images in a within-participants factorial design. All renderings were produced using V-Ray for Rhino (Chaos Group, version 6) under standardized lighting, material, and camera parameters (Figure 2).

Rather than relying on photographic interiors or schematic geometric figures, this designed rendering strategy deliberately brackets extraneous semantic associations while preserving compositional hierarchy, spatial depth, maintaining cues for bodily engagement, and enabling subsequent cross-phase calibration.

Phase 1 employed an online survey administered via Qualtrics (Qualtrics, Provo, UT). The survey instrument was adapted and expanded from established neuroaesthetics protocols. In particular, Chatterjee et al. (2021), building on the interior photograph set introduced by Vartanian et al. (2013) identified four psychologically meaningful affective dimensions of the architectural experience: *pleasure, coherence, fascination, and hominess*. Participants rated each image on a 1–5 scale across core perceptual and evaluative measures, including Liking, Beauty, Curvature, Angularity, Fascination, Coherence, and Hominess (Coburn et al., 2020; Weinberger et al., 2021).

In the present study, additional judgments were collected to contextualize image-based appraisal and to establish observation-linked comparators for subsequent phases. For emotional aspects, participants selected experienced emotions from a predefined list adapted from the Positive and Negative Affect Schedule (PANAS) (Watson et al., 1988). For behavioral aspects, participants estimated the imagined duration of comfortable occupancy (0–200 minutes), judged whether each space appeared more likely to function as *public* or *private*, and indicated regions of visual engagement using a uniform 3 × 3 grid task. Together, these questions constituted a shared survey protocol across all phases and provided reference points within the framework for aligning *reported appraisal* with *observed physiological and behavioral responses*.

Stimuli were presented in randomized order to mitigate sequence effects. Data analysis, conducted in the R environment for statistical computing (R Core Team, 2024), indicated that the intended geometric distinctions were perceptually salient, with substantial inter-participant agreement in ratings of curvature and angularity. Foreground geometry exerted stronger influences on several evaluative dimensions, consistent with the intended figure–ground compositional logic.

A full statistical report of Phase 1 is presented in a separate publication (*Curved background and foreground elements enhance the appeal of indoor spaces*; manuscript in revision). All datasets and materials are publicly available on the Open Science Framework at doi: 10.17605/OSF.IO/B42DC.

### 3.3 Result translation and 3d stimuli refinement

Phase 1 findings serve to assess the stimulus design strategy and to inform the translation of stimuli from 2D image representations to 3D spatial form, guided by two principles: (1) foreground elements are prioritized as the primary carriers of geometric manipulation; and (2) geometric variation is translated from *surface appearance* to *spatial affordance*, shifting the focus from how curvature is visually recognized to how it structures movement, pause, and occupation. This translation yields a *passage–pause–rest schema* as a spatial structuring logic. These modes are employed here as architectural operations rather than semantic labels, describing distinct patterns of bodily engagement: passage as directed movement, pause as transitional slowing or reorientation, and rest as sustained occupation.

Foreground elements from the seven spatial scenarios were re-extracted, decomposed into modular kits, and recomposed into two spatial zones: curved and angular. Each zone measured 3 × 3 × 3 meters and was assembled following the same kit-creation principles described previously, with spatial organization guided by anticipated behavioral flow and the affordances associated with the three engagement modes. The two zones were positioned diagonally within a 3 m (width) × 6 m (length) × 3 m (height) bounding volume to ensure spatial symmetry, formal alignment, and structural balance.

The resulting linear composition was further refined to support build ability and functional clarity. Standardized spatial parameters, such as entry width, ceiling height, and seating elevation, were applied to both zones to preserve analytical comparability while supporting perceptual consistency across conditions.

The outcome, a simplified curvature–angularity continuum named *Suffused Blossom #8*, serves as the basis for both the 1:10 scale prototype in Phase 2 and the full-scale immersive installation introduced in Phase 3.

### 3.4 Prototyping and bridging strategy

In Phase 2, image-based evaluation extends to object-based spatial interaction through the development of a 1:10 scale physical prototype of *Suffused Blossom #8*, thereby enabling geometric properties such as curvature and angularity to be experienced volumetrically rather than pictorially.

The prototype was deployed across multiple institutional settings: an art department, an architecture department, and a public library, thereby extending the diversity of participation. These deployments were conceived as methodological extensions for comparison, allowing the prototype to function as a shared perceptual anchor across disciplinary and public contexts.

**Figure 3:**
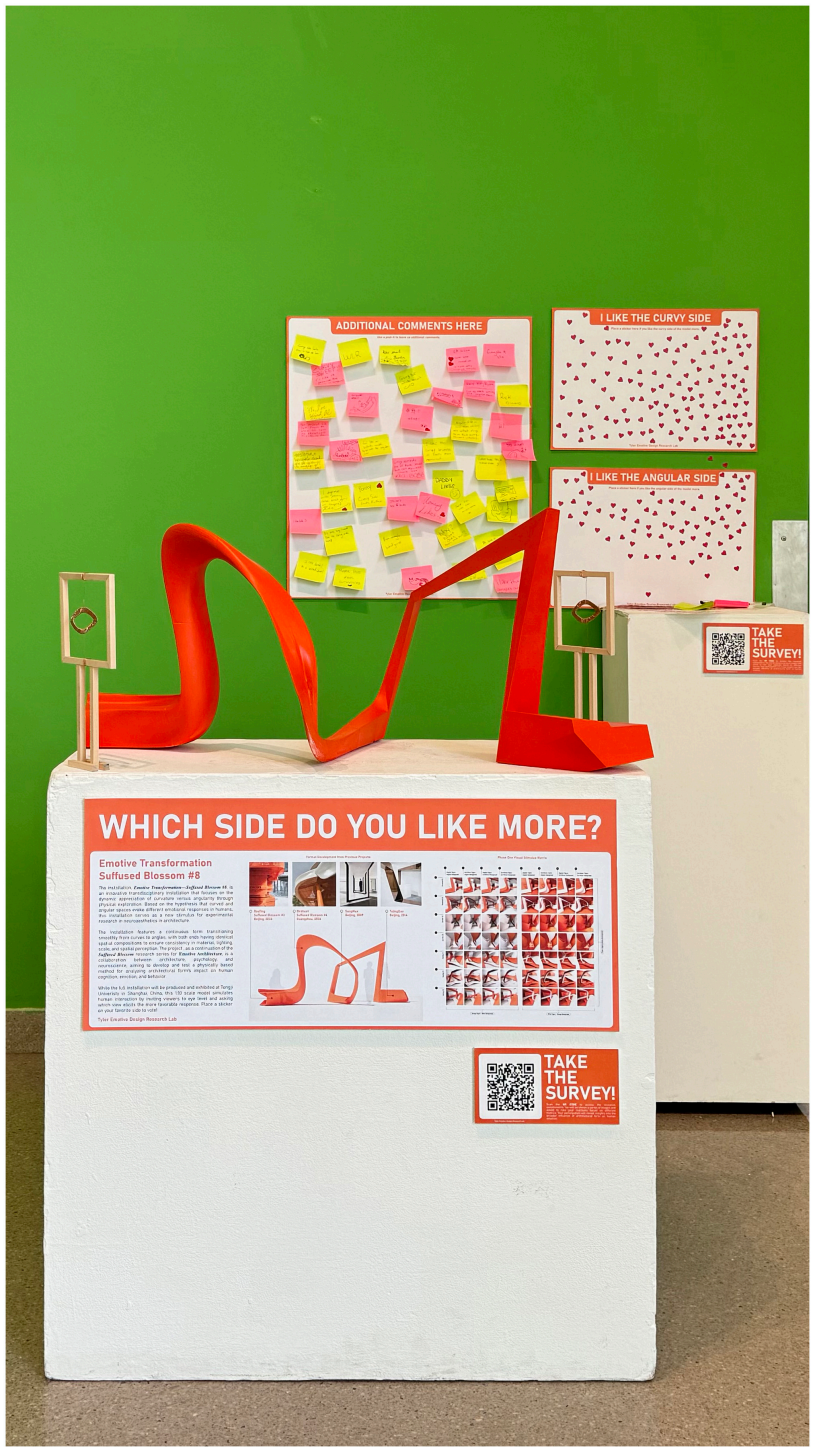
Phase 2 hybrid exhibition as an object-based experimental environment. The 1:10 scale prototype of *Suffused Blossom #8* is presented within an exhibition setting that integrates physical interaction, contextual design information, and participatory evaluation. Beyond serving as a three-dimensional stimulus, the setup provides background on stimulus derivation, supports *in situ* preference voting and open-ended qualitative feedback, and links back to the Phase 1 image-based survey via QR code. This deliberately layered configuration enables investigation of how embodied encounter and contextual knowledge shape subsequent interpretation of two-dimensional image stimuli.

Participants were not subject to time constraints and were encouraged to freely explore the object from multiple viewpoints. Preference assessment was conducted through two complementary procedures. First, participants indicated their immediate spatial preference *in situ* by placing a marker on boards corresponding to either the curved or angular zone of the prototype, capturing intuitive, embodied judgments elicited through direct physical encounter. Second, following engagement with the prototype, participants completed an online survey using the same two-dimensional image stimuli employed in Phase 1 (Figure 3).

By reintroducing the original image set after object-based encounter, Phase 2 functions as a methodological hinge within the framework, testing whether perceptual patterns identified in image-based evaluation remain consistent after the introduction of contextual information. This structure maintains a common evaluative baseline while enabling reflective comparison across representational modes.

**Figure 4:**
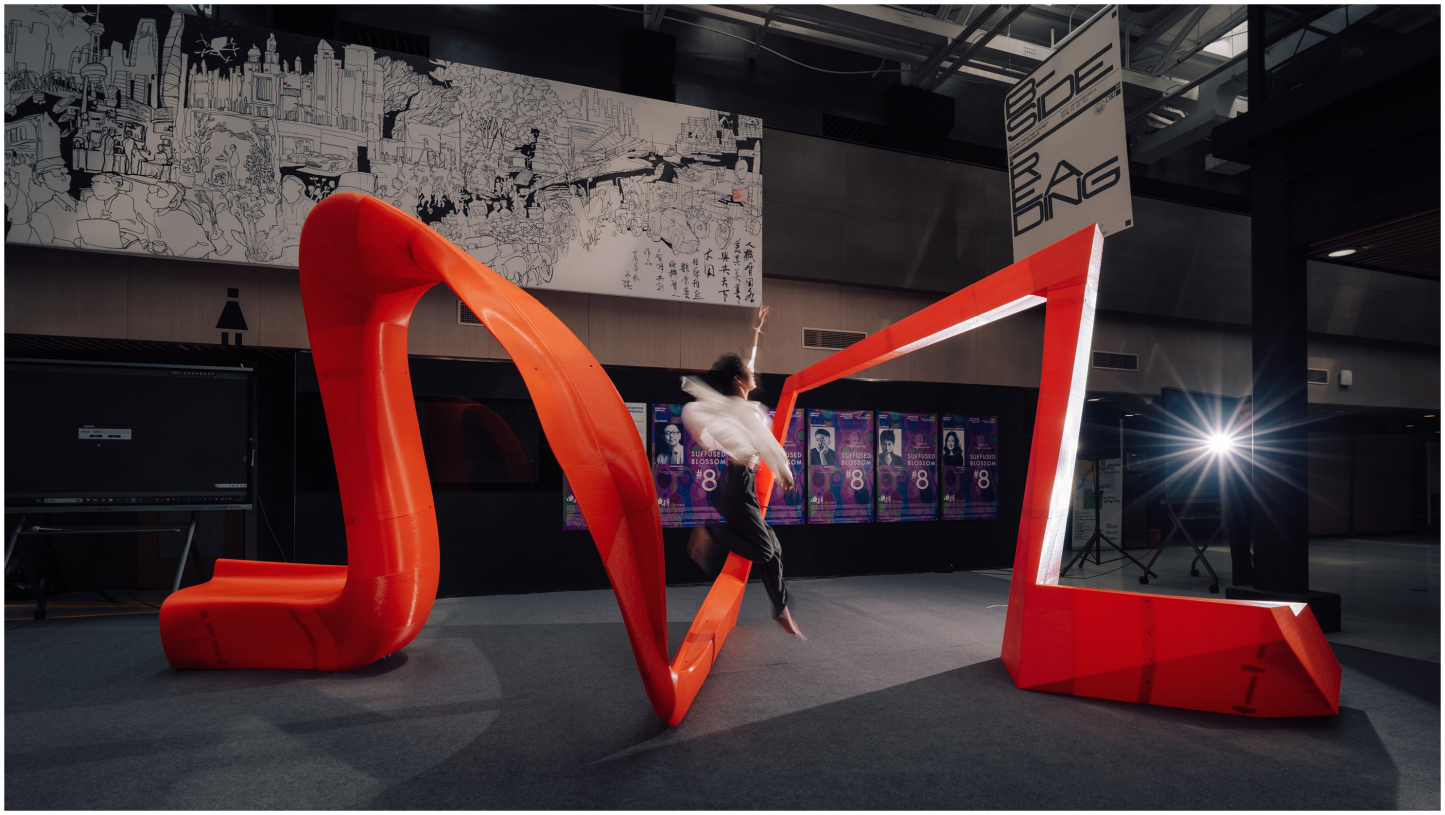
Full-scale installation and emergent embodied interaction during the debut event.The 1:1 realization of *Suffused Blossom #8* presents a continuous curvature–angularity continuum translated from earlier representational phases. During the debut event, dancers, musicians, and visitors—equipped with biosensors—were invited to engage freely with the installation in an immersive setting without predefined tasks. Through this open-ended interaction, a range of embodied behaviors emerged. The photograph presents a dancer's interaction with the central continuous transition between curved and angular regions—originally conceived as part of a unified geometric gradient—that was recurrently engaged as a threshold-like condition, prompting crossing, leaping, and traversal. Such behaviors, visible in the immersive environment, were not anticipated in image-based or object-scale encounters and became apparent only through full-scale, bodily engagement with the spatial continuum.

In Phase 3, *Suffused Blossom #8* was further scaled into a full-scale installation, enabling preference, engagement, and use to be examined under immersive conditions (Figure 4). A detailed account of the design and fabrication process is documented in a separate publication [*Suffused Blossom #8: A Full-Scale Installation Bridging Design and Neuroscience*, accepted conference proceeding forthcoming (2026)].

### 3.5 A platform for future progression

Phase 3 extends this exploratory framework into a fully immersive experimental infrastructure designed to support multimodal investigation of architectural experience across physical and virtual conditions.

Two immersive laboratory settings are employed in parallel. In one setting, participants encounter the full-scale physical installation co-located with a precisely aligned digital twin, enabling experience through a combined physical–immersive condition. In the second setting, participants engage with the same architectural configuration exclusively within a virtual environment. This dual setup enables direct comparison between physical and virtual embodiments of an identical architectural configuration.

Within each setting, participants will be randomly assigned to one of two task conditions: (1) unguided exploration, allowing free movement, path selection, and self-directed dwelling; or (2) directed engagement, prompting navigation toward predefined spatial features. These complementary tasks are designed to elicit patterns of movement, attention, and occupation under contrasting modes of engagement.

Across both settings, the immersive environment functions as an adjustable experimental layer through which contextual parameters—including surrounding environment, lighting, and material appearance—can be systematically varied while preserving geometric consistency. This structure supports comparative analysis of how spatial form interacts with environmental modulation to shape experiential and behavioral responses.

During all task phases, multimodal data will be collected concurrently, including physiological signals via wearable biosensors and behavioral measures such as movement trajectories, dwelling time, and directional choice. Following completion of all immersive tasks, participants will complete an image-based survey using the Phase 1 protocol, with two-dimensional stimuli revised and calibrated to reflect the spatial conditions encountered during laboratory testing. This alignment enables cross-referencing across representational, behavioral, and physiological measures.

Details of the Phase 3 research design and task rationales will be reported in a separate publication. Curvature is treated here as a test case rather than an endpoint, and the Phase 3 platform is structured to support future extensions to additional spatial typologies, functional programs, participant populations, and cultural contexts.

## 4 Conclusion: toward a practice-driven research paradigm

This paper was developed in response to four recurring methodological limitations in prior neuroaesthetics research, and each critique is explicitly mapped onto a corresponding experimental design decision within the proposed framework. To address **stimulus reductionism**, the framework progresses through three representational scales −2D renderings, 3D prototypes, and full-scale immersive environments—thereby reintroducing spatial volume, material articulation, and embodied temporality typically absent from image-based testing. To counter **semantic inconsistency**, all stimuli were derived from a single, coherent architectural corpus, ensuring consistency in design logic, scale, spatial typology, functional context, and figure-ground articulation, with visual contrast calibrated to enhance perceptual clarity. **Fragmented cognition** is mitigated by embedding behavioral affordances –passage, pause, and rest–directly into the spatial compositions, allowing evaluative judgments to emerge from plausible patterns of use rather than abstract visual appraisal. **Ecological validity gap** is addressed by redefining experimentation as an iterative, practice-driven process, in which *perceptual-experiential patterns* emerge through comparison across representational scales and contextual variations, rather than through isolated variable testing. In this framework, experimentation functions not as a shortcut to generalization but as *a generative medium* for engaging the architectural complexity.

Findings from the first experimental phase indicated that stimuli generated through architectural design reasoning can retain perceptual salience while preserving architectural specificity, supporting the viability of design-led stimulus construction within empirical research. More broadly, the framework illustrates that architectural design can operate simultaneously as the object of investigation and the methodological apparatus through which empirical inquiry is structured.

This paper advances a design-integrated experimental framework to the evolving fields of neuroarchitecture with reorienting experimentation away from static hypothesis validation toward an open, iterative research platform for investigating spatial experience.

As with large-scale models in artificial intelligence, whose robustness derives from repeated exposure across diverse environments, the future of neuroarchitecture may depend on the **methodological frameworks capable of scaling** through **situated**, **practice-based inquiry**, allowing patterns to emerge through collective accumulation rather than singular experimental claims.

Ultimately, the strength of this research paradigm lies in its capacity to support distributed, practice-driven experimentation, inviting researchers, designers, and technologists to collaborate not only in measuring experience but in actively shaping the environments through which experience is formed and transformed.

## Data Availability

The datasets presented in this study can be found in online repositories. The names of the repository/repositories and accession number(s) can be found on the Open Science Framework at: doi: 10.17605/OSF.IO/B42DC.
